# Multimodal Postmortem Investigation of Fatal Thoracic Metallic Projectile Trauma in a Jindo Dog: Radiographic, Computed Tomographic, Necropsy, and Histopathological Findings

**DOI:** 10.3390/ani16203263

**Published:** 2026-10-16

**Authors:** Ah-Young Kim, Jongho Kim, Hansung Joo, Sangkyung Cheon, BokKyung Ku, Kyunghyun Lee

**Affiliations:** 1 Division of Animal Diseases Diagnosis, Animal and Plant Quarantine Agency, Gimcheon 39660, Republic of Korea; mochsha@korea.kr (A.-Y.K.); whdgh2339@korea.kr (J.K.); kubk@korea.kr (B.K.); 2 Jukjeon Animal Medical Center, Daegu 42642, Republic of Korea; bettersafe13@gmail.com; 3 Department of Surgical and Radiological Sciences, School of Veterinary Medicine, University of California, Davis, CA 95616, USA; chunsk1987@gmail.com

**Keywords:** veterinary forensic pathology, postmortem computed tomography, projectile injury, gunshot wound, dog, hemopneumothorax, non-accidental injury, forensic virtopsy

## Abstract

Metallic projectile injuries in companion animals present complex diagnostic and evidentiary challenges, especially when comprehensive crime scene data and intact projectiles are absent. This report describes the forensic postmortem investigation of an adult male Jindo dog submitted by law enforcement authorities following fatal penetrating trauma in South Korea. Digital radiography identified multiple radiopaque metallic opacities in the left thoracic region, accompanied by severe pleural effusion and subcutaneous emphysema. Postmortem computed tomography (PMCT; 16-slice MDCT, 0.75-mm slice thickness) precisely delineated clustered hyperattenuating metallic fragments embedded in the left thoracic wall, an oblique displaced fracture of the left fourth rib, marked hemopneumothorax with compression atelectasis, and pneumomediastinum, while excluding cranial or abdominal injury. At necropsy, approximately 100 mL of pleural blood was evacuated, and through-and-through pulmonary transcapsular lacerations were identified traversing the right and left cranial lung lobes. Multiple deformed metallic fragments were recovered adjacent to the fractured rib under strict forensic chain-of-custody protocols. Histopathology of pulmonary laceration margins confirmed acute hemorrhage, fibrin deposition, and early neutrophilic margination, establishing an acute antemortem vital reaction (survival interval < 1 h). Histological examination of the cutaneous entry and exit margins revealed mechanical tearing without thermal coagulation or soot residue, consistent with an intermediate-to-distant discharge. The anatomical convergence of lesions supported a right-to-left projectile trajectory, with the left axillary defect presenting features compatible with a shored exit wound. Death was attributed to fatal acute hemopneumothorax and bilateral pulmonary laceration secondary to perforating metallic projectile injury. This case demonstrates the indispensable diagnostic and prosecutorial value of integrating digital radiography, PMCT virtopsy, forensic necropsy, and histopathology in veterinary medicolegal investigations of non-accidental trauma.

## Simple Summary

Metallic projectile injuries are an important manifestation of traumatic harm in companion animals, often carrying substantial medicolegal implications. In cases of suspected non-accidental injury (NAI) where the weapon, shooting circumstances, and primary ballistic projectile are unavailable, establishing a scientifically defensible cause of death requires a systematic postmortem approach. Here, we report a forensic postmortem investigation of a male Jindo dog with fatal perforating thoracic projectile trauma in the Republic of Korea. By combining digital radiography, high-resolution postmortem computed tomography (PMCT), meticulous forensic necropsy, and histopathological analysis, we successfully localized retained metallic fragments, documented severe pulmonary transcapsular lacerations and left fourth rib fracture, and reconstructed a probable right-to-left trans-thoracic trajectory. This streamlined multimodal workflow highlights how the integration of pre-dissection virtopsy with classical forensic pathology enhances evidence recovery, confirms antemortem vital reaction, and supports legal investigations of suspected animal cruelty.

## 1. Introduction

Metallic projectile injuries are an important cause of traumatic injury in companion animals and may have substantial medicolegal significance [1,2,3,4,5,6]. In recent years, veterinary forensic literature has increasingly focused on the identification and characterization of non-accidental injury (NAI), of which projectile-associated trauma represents a particularly severe and malicious form [1,3,4]. Across international veterinary surveys, gunshot and airgun injuries in dogs and cats are frequently encountered, with thoracic and extremity wounds predominating due to their large surface area and vulnerability during direct encounters [5,6,7]. In jurisdictions with strict firearm and airgun regulations, such as South Korea, civilian firearm ownership is heavily restricted, making domestic projectile injuries relatively uncommon yet legally critical when they occur. Under such legal frameworks, forensic postmortem investigations are essential to determine whether the shooting involved an authorized hunting weapon, an illegal firearm, or a modified airgun, and to distinguish deliberate animal abuse from accidental discharge [1,3,4].

Thoracic penetrating trauma is particularly important since even a relatively focal wound can result in pulmonary transcapsular laceration, hemothorax, pneumothorax, respiratory compromise, and rapid death [5,6]. However, the forensic interpretation of projectile-associated injuries is not always straightforward [3,4,8]. Weapon type, projectile composition, velocity, shooting distance, and exact trajectory may be difficult to establish when the scene has not been fully documented, the main projectile mass is not recovered, or formal ballistic examination is unavailable [7,8,9].

Veterinary forensic pathology presents unique challenges when compared to human forensic medicine. In human forensic investigations, specialized crime-scene units routinely preserve physical evidence, conduct comprehensive ballistics recovery, and document gunshot residues at the scene. In contrast, veterinary practitioners and forensic pathologists are rarely called to crime scenes; instead, deceased animal victims are typically submitted without scene reconstruction or ballistic context [1,2,3,4]. Furthermore, companion animals exhibit dense hair coats that obscure external entrance and exit defects, high soft-tissue elasticity, and extreme anatomical diversity among breeds, which can substantially complicate trajectory reconstruction and wound assessment [3,4]. While human forensic facilities rely routinely on dedicated postmortem virtopsy protocols, access to advanced cross-sectional imaging in veterinary pathology remains limited in many institutions, underscoring the necessity of establishing standardized, reproducible multimodal workflows [2,9,10,11,12,13,14,15].

A multimodal postmortem approach may improve interpretation in such cases [2,7,9,11,13,14]. Conventional digital radiography is useful for rapid detection of radiopaque foreign material and broad characterization of skeletal and thoracic abnormalities [9,11,12]. Postmortem computed tomography (PMCT) provides detailed three-dimensional localization of metallic fragments and permits comprehensive assessment of fractures, intrathoracic gas, fluid accumulation, pulmonary collapse, and subcutaneous emphysema prior to invasive dissection [7,9,11,14]. Pre-necropsy imaging is essential because dissection may inadvertently displace small fragments or disrupt subtle anatomical relationships between skeletal fractures and soft tissue tracts [2,7,9]. Although rare phenomena such as intravascular projectile migration have been reported in the veterinary literature [15,16,17,18], the vast majority of ballistic cases involve direct mechanical penetration and local tissue disruption.

This report describes a fatal case of thoracic perforating metallic projectile injury in an adult male Jindo dog from South Korea. The objective was to integrate digital radiography, PMCT, forensic necropsy, and histopathological analysis to characterize the trauma, evaluate the probable trajectory, elucidate the fatal pathophysiological mechanism, and demonstrate a practical workflow for veterinary medicolegal investigation of suspected non-accidental projectile trauma.

## 2. Materials and Methods

### 2.1. Case Background

A 9.8 kg, intact adult male Jindo dog (estimated age 3–4 years based on complete adult dentition and closed physes) was submitted by local police authorities to the Animal Disease Diagnostic Division of the Animal and Plant Quarantine Agency (APQA) for a forensic postmortem investigation under an official animal cruelty investigation protocol. The carcass was discovered deceased in an outdoor setting and immediately secured by law enforcement. The body was stored refrigerated at 4 °C within approximately 4 h of discovery and submitted for examination within 24 h postmortem. External physical inspection revealed a fresh postmortem condition with established rigor mortis and absent signs of decomposition, autolysis, or putrefactive gas formation, ensuring optimal diagnostic integrity for postmortem virtopsy and necropsy. Available investigative records indicated concern for thoracic projectile injury and possible retained metallic fragments. No scene reconstruction, confirmed firearm identity, caliber, or ballistic reports were initially available.

### 2.2. Postmortem Imaging

Orthogonal thoracic and whole-body radiographs (lateral and ventrodorsal projections) were obtained prior to dissection using a flat-panel digital radiography system (EpiX, POSKOM Co., Ltd., Goyang-si, Republic of Korea) exposure factors: 70 kVp, 10 mAs, focal-film distance 100 cm). Images were reviewed using a DICOM viewing workstation for radiopaque foreign material, skeletal trauma, pleural effusion, and soft tissue emphysema.

Subsequently, postmortem computed tomography (PMCT) was performed with a 16-slice multi-detector CT scanner (Brivo CT385, GE Healthcare, Beijing, China). Helical volumetric data were acquired in dorsal recumbency (120 kVp, 150 mAs, slice thickness 0.75 mm, reconstruction increment 0.5 mm, pitch 0.938, gantry rotation time 0.8 s). Both high-resolution bone kernel and standard soft-tissue reconstruction algorithms were processed and archived. Multiplanar reconstructions (transverse, sagittal, dorsal) and three-dimensional (3D) volume rendering were performed using dedicated DICOM software version 14BW25.15. (OsiriX MD, Pixmeo, Geneva, Switzerland; window width 2000 HU, window level 500 HU for bone; WW 400 HU, WL 40 HU for soft tissue; WW 1500 HU, WL −600 HU for lung). CT review focused on metal attenuating fragment localization as well as detailed evaluation of thoracic soft tissue and osseous structures and the presence or absence of traumatic lesions in the head, spine, and abdomen.

### 2.3. Necropsy and Sample Collection

A complete, systematic layer-by-layer forensic necropsy was conducted following established veterinary forensic evidence collection protocols [2,4,13]. Prior to incising the thoracic wall, the presence of pneumothorax and loss of thoracic negative pressure were evaluated by performing a submerged diaphragmatic puncture test (‘water-test’) under direct visualization. External cutaneous defects were photographed, measured, and documented. Dissection carefully followed the roadmap provided by the observed imaging findings to expose disrupted skeletal structures and recover retained metallic fragments. Following the formal chain-of-custody documentation, all recovered metallic fragments were documented in situ, rinsed gently in saline without mechanical scraping, weighed, photographed with a forensic scale, and transferred to the National Forensic Service [2,13].

Tissue samples from the margins of internal pulmonary parenchymal injuries, cutaneous entry and exit lesions, subcutaneous tissue, and adjacent intercostal musculature were fixed in 10% neutral-buffered formalin, embedded in paraffin wax, sectioned at 4 µm, and stained with hematoxylin and eosin (H&E). Histopathological examination evaluated hemorrhage, fibrin deposition, cellular inflammatory response, and microscopic evidence of thermal injury or foreign particulates.

## 3. Case Presentation

### 3.1. External Examination

**Figure 1:**
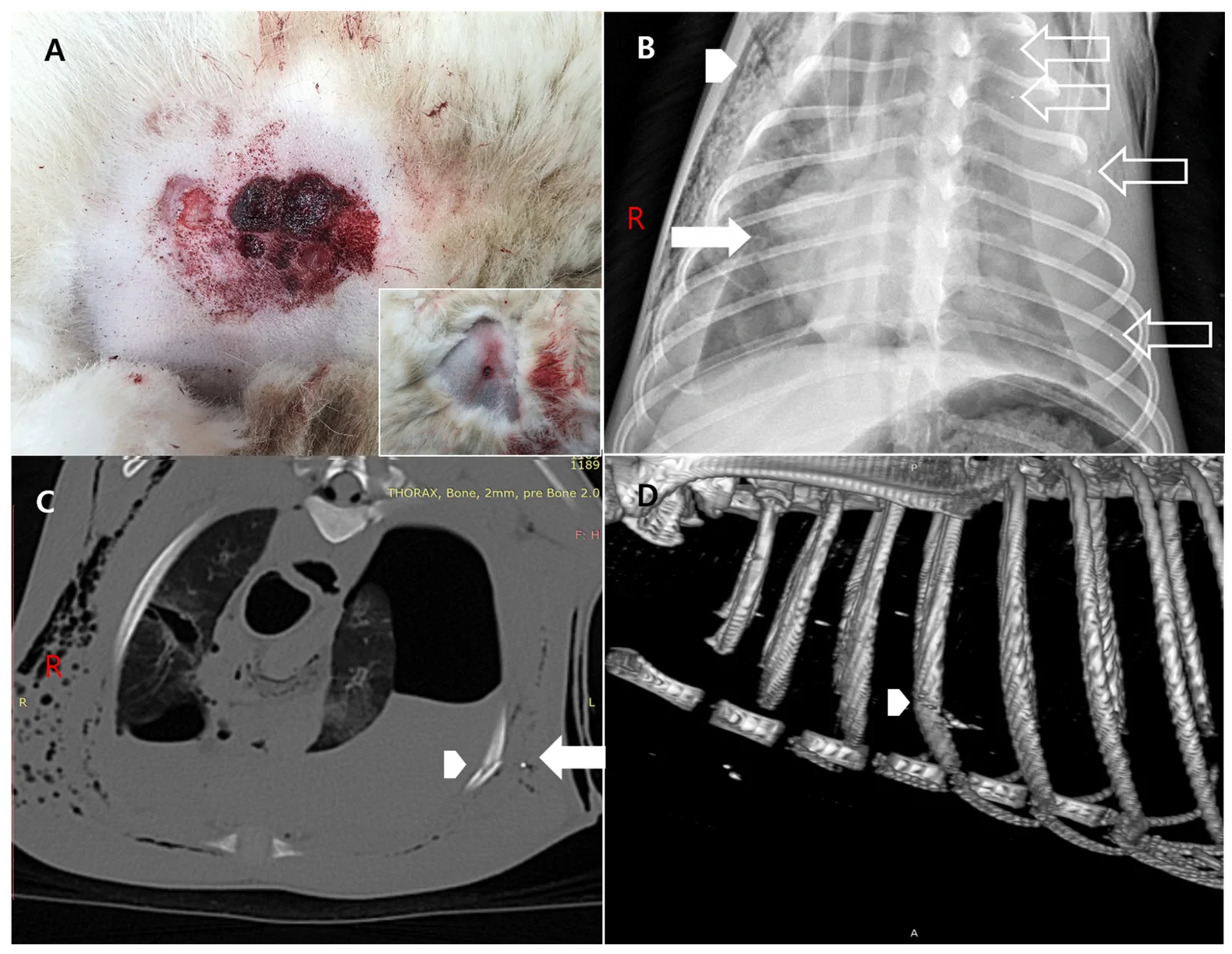
External, radiographic, and postmortem computed tomographic findings in a dog with fatal thoracic metallic projectile trauma. (**A**) External view of the left axillary irregular exit defect with adjacent abraded margin compatible with a shored exit wound and the right scapular circular entry defect (inset). (**B**) Ventrodorsal (VD) thoracic radiograph demonstrating multiple radiopaque metallic opaque fragments (open arrows) clustered in the left hemithorax, marked pleural effusion obscuring the cardiac silhouette (arrow), and left thoracic subcutaneous emphysema (arrowhead). (**C**) Transverse PMCT image (bone window: WW 2000, WL 500; sharp reconstruction kernel) showing an oblique displaced fracture of the left fourth rib (arrowhead) with adjacent clustered hyperattenuating metallic fragments (arrow), left-sided pneumothorax with right mediastinal shift indicating tension pneumothorax, and compression atelectasis. (**D**) 3D volume-rendered CT reconstruction of the thoracic cage illustrating the displaced left fourth rib fracture (arrowhead) and spatial distribution of metallic fragments along the left thoracic wall.

External examination revealed an intact hair coat with focal blood-staining on the right shoulder and left axilla. Following clipping of hair, a discrete circular skin defect measuring 6 mm in diameter was identified over the right scapular region, surrounded by a faint circumferential abrasion collar (Figure 1A). In the left axillary region, an irregular, slightly elongated, stellate skin defect measuring approximately 14 mm × 9 mm was observed, accompanied by an eccentric zone of superficial epidermal abrasion along its caudoventral margin, morphologically compatible with an exit wound with possible shoring (Figure 1A). Palpation revealed marked crepitus over the left lateral thoracic wall consistent with subcutaneous emphysema. No evidence of contusion, laceration, or skeletal fracture was identified in the head, spine, pelvis, or extremities.

### 3.2. Radiographic Findings

Orthogonal thoracic radiographs revealed multiple (6–8) discrete, irregularly shaped, sharply marginated metallic opacities ranging from 1.0 mm to 3.5 mm in maximal diameter, clustered primarily in the left ventral and mid-thoracic region projecting over the left 4th to 6th intercostal spaces (Figure 1B). A moderate-to-large volume of pleural effusion was evident, characterized by bilateral scalloping of lung lobes, dorsal retraction of pulmonary margins, and generalized soft-tissue opacity obscuring the ventral cardiac silhouette and diaphragmatic line. Thoracic subcutaneous emphysema was marked along the left lateral body wall. Retrospective evaluation of the lateral projection demonstrated an irregular cortical discontinuity and step-off deformity at the mid-shaft of the left fourth rib, partially obscured by forelimb musculature.

### 3.3. Postmortem Computed Tomographic Findings

PMCT provided comprehensive three-dimensional mapping of the thoracic trauma. An oblique, displaced fracture of the left fourth rib was identified at its mid-to-dorsal third, with outward (lateral) displacement of the cortical margins. Clustered hyperattenuating metallic fragments (CT attenuation > 3000 HU) were embedded within the disrupted intercostal musculature and parietal pleura immediately adjacent to the fractured rib (Figure 1C,D). Additionally, a moderate volume of bilateral pleural effusion (CT attenuation ranging from 35 to 50 HU, consistent with unclotted to partially clotted blood) was documented, and several tiny metallic fragments (1–2 mm) had migrated into the dependent aspect of the left costodiaphragmatic recess within accumulated pleural fluid. Marked left-sided pneumothorax was identified, accompanied by extensive thoracic subcutaneous emphysema along the left thoracic wall, as well as a prominent mediastinal shift to the contralateral (right) side indicating left-sided tension pneumothorax. The left lung lobes showed severe passive compression atelectasis, while the right lung lobes exhibited patchy alveolar consolidation compatible with contusion and hemorrhage.

Careful examination of the cranial mediastinum revealed focal pneumomediastinum and mild mediastinal widening with soft tissue attenuation along the prospective right dorsal-to-left ventral trans-thoracic tract connecting the cranial lobes. However, the major mediastinal vascular structures—including the cranial vena cava, aortic arch, brachiocephalic trunk, and left subclavian artery—remained intact with preserved wall continuity and no evidence of transection or pseudoaneurysm. Adjacent organs including the esophagus, trachea, heart, and pericardium demonstrated no signs of traumatic rupture, blunt contusion, or temporary cavitation injury. Whole-body CT survey confirmed the complete absence of traumatic or pathological abnormalities in the calvarium, brain parenchyma, cervical/thoracolumbar vertebrae, and abdominal viscera.

### 3.4. Necropsy Findings

Diaphragmatic puncture under water submersion yielded immediate release of pressurized gas bubbles, confirming the presence of pneumothorax prior to opening the pleural cavity. Upon thoracic entry, approximately 100 mL of dark red, liquid to partially clotted blood (hemothorax) was evacuated from the thoracic cavity, with marked accumulation on the left side.

Detailed dissection of the pulmonary parenchyma revealed distinct focal transcapsular laceration tracts. In the right cranial lung lobe, a perforating defect entered the lateral surface (defect 8 mm × 6 mm) and exited the medial aspect (9 mm × 7 mm), surrounded by dark hemorrhagic parenchymal cuffs. In the left cranial lung lobe, a corresponding through-and-through transcapsular laceration (entry 9 mm × 8 mm, exit 12 mm × 10 mm) traversed the ventral third, presenting ragged, hemorrhagic margins. The wound trajectory formed a direct anatomical continuum across the ventral cranial mediastinum located ventral to the major vascular structures, connecting the right entry site to the left thoracic wall defect.

On the inner surface of the left thoracic wall, severe tearing of the fourth intercostal artery, vein, and parietal pleura was present adjacent to the fractured left fourth rib, representing the primary source of the hemothorax, alongside bleeding from the lacerated cranial lobar pulmonary vessels. Extensive intramuscular and fascial hemorrhage involved the left axillary and pectoral musculature directly underlying the left axillary skin defect. Careful dissection of the fractured rib and perilesional soft tissues yielded five deformed metallic fragments (aggregate weight 1.78 g), which were cataloged and preserved for forensic ballistics. No large intact projectile core was identified within the carcass.

Detailed inspection of the right internal thoracic wall revealed focal laceration and extensive muscular hemorrhage within the right third and fourth intercostal spaces surrounding the entry tract; however, the right intercostal neurovascular bundles remained grossly intact without major arterial transection, indicating that right hemithoracic fluid primarily originated from the lacerated right pulmonary parenchyma and associated capillary bed.

### 3.5. Histopathological Findings

**Figure 2:**
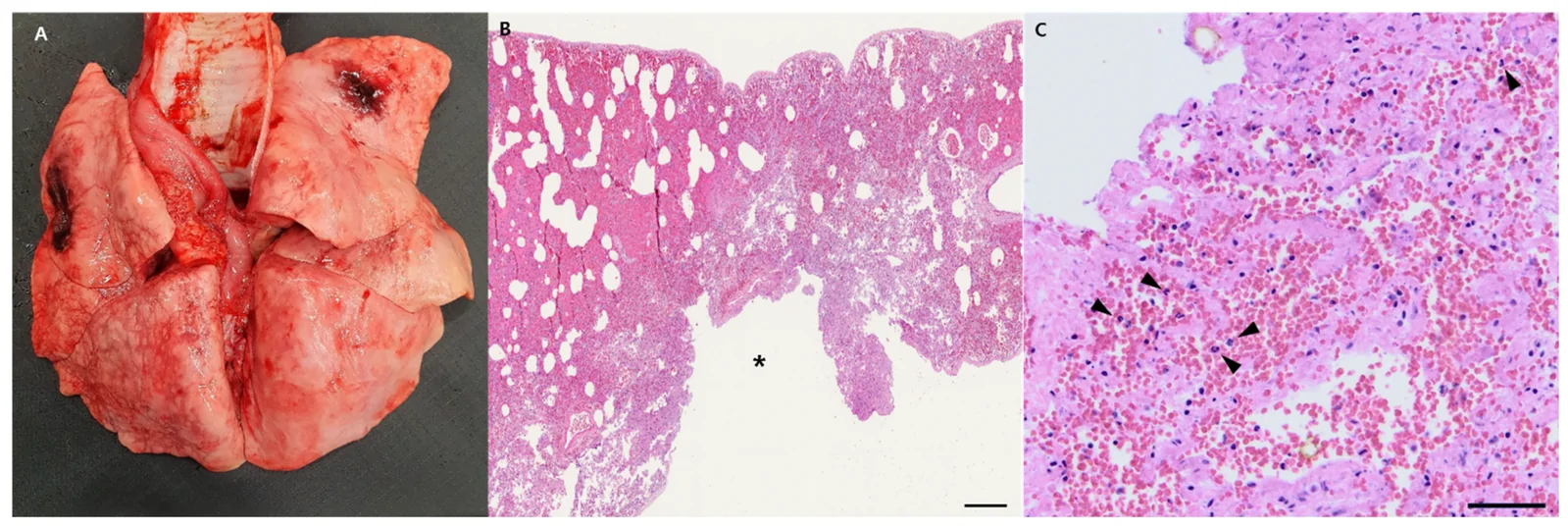
Gross and histopathological pulmonary and cutaneous lesions associated with thoracic projectile trauma. (**A**) Gross necropsy view of through-and-through transcapsular lacerations involving the right cranial lung lobe and left cranial lung lobe accompanied by acute parenchymal hemorrhage. (**B**) Photomicrograph of the left cranial pulmonary laceration margin (*) demonstrating extensive alveolar rupture and extravasated erythrocytes (H&E, 40× magnification, scale bar = 500 µm). (**C**) Fibrin meshwork deposition and infiltrating neutrophils (arrowheads) in the hemorrhagic lesion supported an acute antemortem vital reaction (H&E, 400× magnification, scale bar = 50 µm).

Histopathological examination of the margins of the pulmonary transcapsular lacerations revealed extensive disruption of alveolar architecture, massive alveolar and interstitial erythrocyte extravasation, dense fibrin meshwork deposition, and early marginating and infiltrating neutrophils within adjacent capillaries and perivascular spaces (Figure 2B). Fibroblastic proliferation, mature collagen deposition, and hemosiderin-laden macrophages were entirely absent. These findings confirmed an acute antemortem vital reaction, indicating that the dog was alive when the projectile traversed the lungs, with survival estimated at between several minutes and less than one hour.

Histological evaluation of the right scapular skin defect revealed epidermal and dermal mechanical perforation, collagen bundle tearing, and acute dermal hemorrhage. Importantly, there was no epidermal coagulation necrosis, epithelial heat distortion, or dermal carbon/gunpowder particulate deposition. Sections of the left axillary exit defect showed full-thickness epidermal laceration and subcutaneous hemorrhage without thermal changes. These histological characteristics confirm mechanical projectile trauma without close-range or contact thermal-blast phenomena.

## 4. Discussion

This case demonstrates the complementary diagnostic value of integrating digital radiography, PMCT, forensic necropsy, and histopathology in the medicolegal evaluation of fatal projectile trauma in a companion animal(Table 1) [1,2,3,4,7,9,11,13,14]. In veterinary forensic medicine, projectile-induced trauma often occurs in the context of non-accidental injury (NAI) or suspected animal cruelty, requiring examiners to provide an objective, scientifically defensible determination of the cause of death and reconstruction of traumatic events [1,3,4]. When investigative circumstances lack firearm recovery or ballistic verification, a rigorous multimodal postmortem approach provides the essential evidentiary bridge between physical lesions and legal interpretation.

PMCT virtopsy served as an indispensable pre-dissection tool in this investigation. As previously emphasized in both human and veterinary forensic literature [2,7,9,10,13,14], conventional necropsy alone carries inherent risks of inadvertently altering small metallic fragment positions, creating artificial tissue tracts, or obscuring subtle bone fractures during rib cage reflection. By conducting thin-section helical PMCT prior to opening the thoracic cavity, we mapped the precise anatomical distribution of metallic fragments—distinguishing fragments embedded in the left fourth intercostal musculature from those resting gravitationally in the dependent pleural recess—and documented the displaced oblique rib fracture. Furthermore, PMCT enabled a non-invasive survey of the entire carcass, confirming the complete integrity of the neurocranium, spine, and abdomen, thereby excluding concurrent traumatic or pre-existing debilitating pathology [2,7,9,14].

A critical pathophysiological question in forensic traumatology concerns the precise mechanism of death. In clinical veterinary emergency practice, traumatized dogs with isolated blunt or penetrating chest trauma frequently present with small-to-moderate hemothorax or pneumothorax and can achieve excellent survival rates with tube thoracostomy and conservative therapy [5,6]. A hemothorax volume of approximately 100 mL in a 9.8 kg dog represents approximately 12% to 15% of total circulating blood volume (calculated at ~80–90 mL/kg), which alone would induce moderate compensatory hypovolemia rather than immediate exsanguination [5,6]. However, in the present case, necropsy and PMCT demonstrated the catastrophic synergy of concurrent bilateral pulmonary transcapsular lacerations and left-sided tension hemopneumothorax. The pressurized accumulation of pleural air (confirmed by positive underwater diaphragmatic puncture) and blood caused severe compression atelectasis of the left lung lobes, profound mediastinal shift, and substantial ventilation-perfusion mismatch. Concurrently, elevated positive intrathoracic pressure collapsed the cranial and caudal vena cava, drastically diminishing cardiac venous return and precipitating acute obstructive circulatory shock and rapid respiratory arrest. This multi-factorial failure explains the acute fatal outcome.

**Figure 3:**
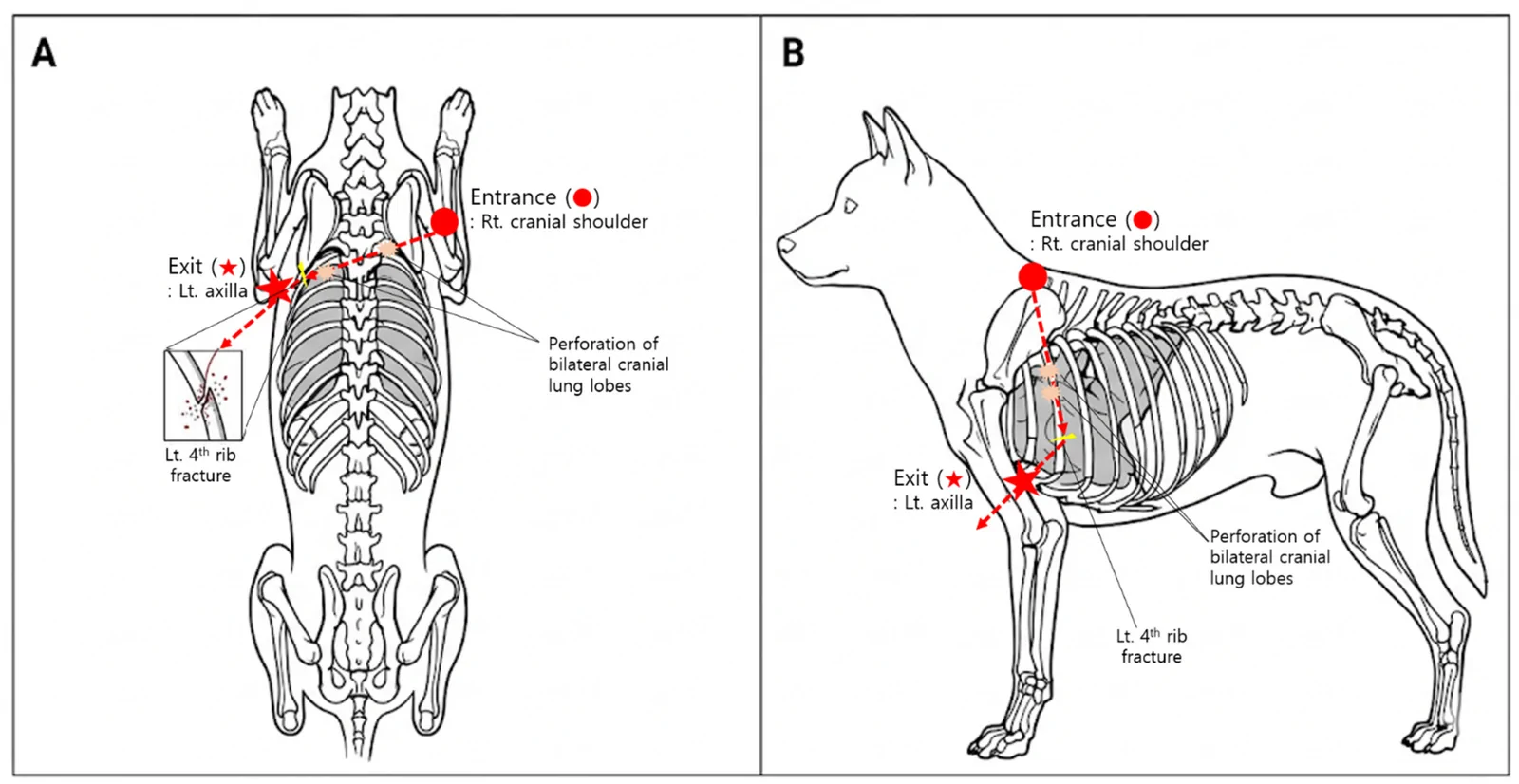
Schematic reconstruction of the probable thoracic projectile pathway. (**A**) Dorsal schematic view illustrating the right-to-left trans-thoracic trajectory originating from the right scapular entry defect (6 mm), traversing the right and left cranial lung lobes through the cranial mediastinum, impacting the left fourth rib, and terminating at the left axillary exit defect (14 mm). (**B**) Left lateral schematic view showing the spatial relationships among the pulmonary lacerations, outward displaced left fourth rib fracture, and exit skin lesion.

Reconstruction of the projectile pathway is another paramount objective in forensic analysis [3,4,8,17]. The convergence of physical and imaging findings in this dog establishes a highly defensible right-to-left trans-thoracic trajectory: (1) an initial entry defect on the right scapular region displaying a small circular diameter (6 mm) with an abrasion ring; (2) continuous transcapsular laceration tracts traversing the right cranial lung lobe and left cranial lung lobe across the cranial mediastinum; (3) direct impact and outward displacement of the left fourth rib mid-shaft; (4) localized fragmentation and retention of metallic debris along the left thoracic wall; and (5) an irregular, larger (14 mm) exit-type defect in the left axilla (Figure 3). The left axillary skin defect displayed features compatible with an exit wound with possible shoring, likely caused by the dog collapsing into left lateral recumbency or the axilla being supported by ground/substrate at the moment of projectile emergence [3,4,8]. The outward beveling and displacement of the left fourth rib fragments provide conclusive anatomical confirmation that the projectile struck the inner cortical aspect of the rib from within the thoracic cavity, moving from medial to lateral [3,8].

The absence of temporary cavitation injury or remote parenchymal contusion in adjacent mediastinal structures (esophagus, trachea, and great vessels) is a notable ballistic finding. In high-velocity rifle injuries, expansive temporary cavitation produces widespread parenchymal tearing, micro-fractures, and extensive vascular ruptures distant from the permanent tract [3,8]. In this case, the highly localized permanent cavity and lack of collateral cavitation damage indicate that the projectile was either a low-to-medium-velocity projectile (such as a handgun bullet or high-power airgun slug) or had significantly decelerated before entering the thoracic cavity, expending its residual kinetic energy primarily during impact with the left fourth rib [3,4,8]. Furthermore, histopathological examination of the entry cutaneous margins showed an absence of dermal heat necrosis, epithelial scorching, or soot particles, definitively excluding a contact or close-range gunshot and indicating an intermediate-to-distant discharge [3,8].

Differential diagnoses for penetrating thoracic trauma in domestic animals include sharp-force injury (e.g., stabbing with knives, spears, arrows, or crossbow bolts) and dog-bite lacerations [3,4,9]. Sharp implements produce clean, linear incision margins, lacking circumferential abrasion collars, projectile fragmentation, or scattered metallic debris embedded in bone [9]. Bite trauma typically presents with paired ‘crush-tear’ punctures, canine tooth spacing patterns, and extensive subcutaneous avulsion [3,4]. The presence of fragmented hyperattenuating metallic material on PMCT, confirmed by necropsy recovery of metallic shards, firmly rules out impalement or animal bites and substantiates metallic projectile trauma [3,4,8].

Histopathological evaluation provided vital confirmatory evidence. The detection of fibrin networks, extravasated erythrocytes, and early marginating neutrophils within pulmonary laceration borders established an indisputable antemortem vital reaction [1,2,4]. The absence of fibroplasia, endothelial sprouting, or hemosiderin deposits indicates that the survival time was brief—likely within minutes to less than an hour post-injury. This microscopic timeline corroborated that the fatal collapse was directly attributable to the acute ballistic trauma rather than secondary infectious or delayed complications.

In companion animal gunshot case series (such as those reported by Olsen et al. [6], Capak et al. [5], and Grela et al. [7]), thoracic gunshot trauma accounts for 20% to 35% of presentations, carrying the highest mortality rate among anatomical regions due to rapid hemopneumothorax and cardiopulmonary arrest. The present case represents a classic yet forensically intricate example of fatal perforating thoracic trauma, illustrating the necessity of standardizing forensic virtopsy in veterinary medicine [2,7,11,13,14]. Collaboration between veterinary pathologists, veterinary radiologists, and forensic ballistics experts is paramount to advance the prosecution of animal cruelty and enforce animal welfare legislation.

## 5. Conclusions

This report documents a fatal thoracic perforating metallic projectile injury in a Jindo dog investigated through an integrated multimodal approach. Digital radiography, PMCT virtopsy, systematic forensic necropsy, and histopathology converged to establish an acute antemortem right-to-left trans-thoracic trajectory with pulmonary transcapsular lacerations, left fourth rib fracture, left thoracic wall metallic fragmentation, and fatal hemopneumothorax. PMCT proved crucial in mapping metallic fragments, evaluating skeletal integrity, and documenting life-threatening tension hemopneumothorax without disturbing physical evidence. Histopathological findings confirmed an acute vital reaction (<1 h survival) and excluded contact-range thermal discharge. This comprehensive workflow highlights the growing importance of veterinary forensic virtopsy and pathology in supporting legal investigations of suspected animal abuse and non-accidental trauma.

## Figures and Tables

**Table 1:** Summary of postmortem diagnostic imaging findings and their pathologic correlations at necropsy in a dog with fatal thoracic perforating projectile trauma.

Diagnostic Modality	Key Imaging/Diagnostic Findings	Pathologic Correlation at Necropsy
Digital Radiography (VD & Lateral)	Clustered radiopaque metallic opacities (left 4th–5th ICS); marked pleural effusion; subcutaneous emphysema; left 4th rib fracture step-off.	Deformed metallic fragments; ~100 mL hemothorax; soft tissue gas tracking along fascial planes; mid-shaft left 4th rib fracture.
Postmortem CT (PMCT)	Hyperattenuating metallic fragments embedded in left thoracic wall and dependent costodiaphragmatic recess; left 4th rib oblique fracture; hemopneumothorax with contralateral mediastinal shift; pneumomediastinum; intact large vessels.	Osseous impact and fragmentation; left intercostal and pulmonary vascular lacerations; cranial lobar transcapsular tracts; intact aorta and vena cava.
Gross Necropsy	Pneumothorax confirmed by water test; through-and-through transcapsular lacerations in right and left cranial lung lobes; left axillary exit wound with possible shoring.	Direct right-to-left trans-thoracic projectile pathway; outward rib fracture displacement; 5 recovered metallic fragments (1.78 g); absent cavitation in adjacent organs.
Histopathology (H&E)	Fibrin strands, extravasated erythrocytes, and early neutrophilic margination at pulmonary laceration edges; mechanical dermal rupture without thermal necrosis or soot.	Confirmation of acute antemortem vital reaction (<1 h survival); exclusion of contact/close-range thermal discharge; differentiation from postmortem damage.

## Data Availability

The original contributions presented in this study are included in the manuscript. Further inquiries can be directed to the corresponding author. The data supporting the conclusions of this article may be made available by the corresponding author upon request, subject to institutional guidelines and a data use agreement.
